# The Impact of Psychosocial Interventions on Older Adults in the Community Experiencing Social Isolation: An Integrative Review

**DOI:** 10.1111/inm.70186

**Published:** 2025-12-02

**Authors:** Ann‐Marie Keane, Ann‐Marie Bright, Luca Greenan, Annmarie Grealish

**Affiliations:** ^1^ School of Nursing and Midwifery Health Research Institute, University of Limerick Limerick Ireland; ^2^ Florence Nightingale Faculty of Nursing, Midwifery & Palliative Care King's College London London UK

**Keywords:** community mental health, mental health, older adults, psychosocial interventions, social isolation

## Abstract

The older adult population aged 65 years and over is growing rapidly and social isolation among this population has been recognised as a significant public health concern. High rates of physical, emotional and cognitive comorbid conditions are commonly linked among older adults with social isolation. As the older adult population increases, the need for psychosocial interventions will increase in order for health services to serve this ageing population. The aim of this review was to explore and evaluate the effectiveness of psychosocial interventions implemented in community settings to reduce social isolation in older adults. Whittemore and Knafl's five‐stage integrative review framework guided this review as it facilitates the inclusion and integration of diverse methodological approaches to experimental research. Five electronic databases (CINAHL, Medline, Web of Science, PsycINFO and Embase) were systematically searched and reported according to the Preferred Reporting Items for Systematic Reviews and Meta‐Analyses guidelines. Of the 14 989 studies retrieved from database search, seven studies met the inclusion criteria and were synthesised using narrative synthesis. Ten types of psychosocial interventions for older adults with social isolation were identified that can be delivered by healthcare professionals. The positive impact of these on social isolation, and comorbidities such as depression and anxiety has been demonstrated. In addition, we identified the useful components of these interventions and the experiences of older adults and healthcare professionals in delivering psychosocial interventions, thereby highlighting the key elements that contribute to successful outcomes. Findings strongly suggest the need for enhanced support structures and greater integration of psychosocial approaches into routine community care for older adults. Therefore, training for healthcare professionals to provide psychosocial interventions to older adults in the community is needed.

## 1 Introduction

The older adult population aged 65 years and over is growing rapidly and is estimated to have increased by more than 40% in the past decade (World Health Organization (WHO) 2024). Globally, by 2050, the number of people aged 65 years and older is expected to double to 1.6 million and the number of people aged 80 years and older is expected to triple to 426 million (WHO 2024). As this population increases, the need for psychosocial interventions (PSIs) and behavioural health services will also increase (Min et al. 2023; Nagel et al. 2023). Social isolation and loneliness among older adults have been recognised as significant public health concerns (WHO 2021; Centres for Disease Control and Prevention (CDC) 2024), with growing evidence linking this population to adverse physical, emotional and cognitive health outcomes (Holt‐Lunstad et al. 2015; National Academies of Science, Engineering and Medicine (NaSEM) 2020; CDC 2024). Social isolation is commonly defined as a lack of social contact or engagement with others (Nicholson 2009). According to Cornwell and Waite (2009), it is an objective condition characterised by minimal interactions, limited participation in social activities and a small number of social roles. Loneliness on the other hand, refers to the subjective experience of distress stemming from perceived social disconnection (Qualter et al. 2023). Tzouvara et al. (2015) describe loneliness as the emotional discomfort that arises when there is a gap between an individual's desired and actual social relationships, highlighting both emotional and cognitive dimensions.

Although these terms are often used interchangeably, they represent distinct but interrelated experiences that can significantly affect wellbeing (Coyle and Dugan 2012; Valtorta et al. 2016). Whilst this review acknowledges the overlap between social isolation and loneliness, its focus is specifically on social isolation due to its unique risk factors and broader implications for physical and mental health. Recent data suggest that approximately one in four older adults experience social isolation, with individuals who live alone, those who manage chronic illness, and those who experience functional decline being particularly at risk (Nicholson 2012; Cadjoe et al. 2020; NASEM 2020; WHO 2021).

In the Australian context, it is estimated that 15% of individuals experience social isolation, with males being more severely affected than females (Australian Institute of Health and Welfare (AIHW) 2025). Indeed, loneliness in the United States is described as an ‘epidemic’ (pg. 1) where the highest rates of social isolation are found in those aged 65 and over (Department of Health and Human Services 2023). In Ireland, it is estimated that around 16% of adults aged 65 and older experience social isolation based on objective measures of social contact (The Irish Longitudinal Study on Ageing (TILDA) 2014). In the UK, estimates suggest that 7% of older adults are socially isolated, with increased risk among those who live alone, those who have mobility issues and those who lack community supports (Age UK 2023; Office for National Statistics (ONS) 2023).

## 2 Background

The WHO (2021) has highlighted a strong association between social isolation and adverse health outcomes, including a 50% increased risk of dementia and a 29% increased risk of heart disease. This is supported by recent research from Johns Hopkins University, which found that socially isolated older adults had a 27% higher risk of developing dementia over a nine‐year period compared to their socially connected peers (Shaurya et al. 2023). Similarly, the American Heart Association (AHA) reports that social isolation and loneliness are associated with a 29% increased risk of heart attack or death from heart disease and a 32% higher risk of stroke (Cené et al. 2022).

Additionally, social isolation significantly elevates the risk of suicide among older adults, particularly men over the age of 75 (CDC 2021; National Council on Ageing 2023) for whom global suicide rates are estimated to be more than double the average (WHO 2021). Social isolation has been identified as a major predictor of suicidal ideation and behaviour in older populations (Vanderhorst and McLaren 2005; Calati 2019). Courtin and Knapp (2017) note that socially isolated individuals are more prone to feelings of hopelessness and a diminished sense of purpose, both well‐established risk factors for suicide. Pusey et al. (2005) also identified that older adults with depression, who engage in self‐harming behaviours, often have fewer social resources and support networks, reinforcing the role that social connection plays in protecting mental health and preventing suicide in this demographic. Recent studies by McClelland et al. (2020) and Ward et al. (2024) also highlighted how social disconnection is not only linked to heightened psychological distress but also contributes to elevated mortality risk through suicide‐related pathways.

There is well‐established co‐morbidity between social isolation and mental health such as depression and anxiety (Cacioppo et al. 2006; Santini et al. 2015; Zhang et al. 2023). These studies have shown how socially isolated individuals are significantly more likely to experience symptoms of both depression and anxiety, with persistent isolation often worsening existing mental health challenges. This cycle places growing pressure on health and social care systems due to the increased need for mental health services, crisis intervention and hospitalisation related to depression, self‐harm and suicide risk (Perissinotto and Covinsky 2023; Ward et al. 2024). Social isolation has also been linked to increased mortality risk, comparable to established public health risks such as obesity and smoking (Steptoe et al. 2013; U.S. Department of Health and Human Services 2023). It has also been identified that social isolation can increase the risk of early death by up to 29%, reinforcing the urgency of developing effective, evidence‐based solutions to this issue (Pantell et al. 2013; Holt‐Lunstad et al. 2015; Nakou et al. 2018, 2025; Xia and Li 2018).

The definition of PSIs applied to this review is a group of non‐pharmacological therapeutic interventions grounded in psychological theory designed to promote psychological, social and interpersonal relationships (Dua et al. 2011; Barbui et al. 2020). PSIs have gained momentum as a key method for addressing social isolation among older adults by encouraging meaningful connections, improving mental health and enhancing overall quality of life (Dickens et al. 2011; Hoang et al. 2022; Verity et al. 2023). PSIs can take many forms, including peer support groups, community‐based activities, reminiscence therapy and cognitive behavioural interventions. Multi‐modal interventions that combine group activities with one‐to‐one support have been shown to be especially effective in addressing the complex and layered nature of social isolation (Gardiner et al. 2016; Hoang et al. 2022; Phang et al. 2023).

Despite the growing application of PSIs (Gardiner et al. 2020; Hoang et al. 2022), questions remain about their overall effectiveness. Differences in intervention design, delivery formats and outcome measurement have made it difficult to identify which approaches consistently provide the best results (Hunter et al. 2016; Gardiner et al. 2018; Williams et al. 2021; Hoang et al. 2022). Although some research suggests that group‐based interventions can enhance social connectedness through shared activities and peer support (Cattan et al. 2005; Dickens et al. 2011), others point to the value of more personalised approaches such as individual counselling and mentoring which may address deeper psychological barriers to engagement (Windle et al. 2011; Fakoya et al. 2020). Successful interventions often offer flexibility in delivery methods, including both face‐to‐face and virtual options, to accommodate different levels of need and access (Noone et al. 2020). Interventions such as cognitive behavioural therapy (CBT), reminiscence therapy and peer‐led programmes have been shown to improve social participation, reduce depressive symptoms, enhance self‐esteem and increase perceived support (Masi et al. 2010; Gardiner et al. 2016; Woods et al. 2018). However, despite promising findings, questions remain about which specific interventions are most effective, for whom, and in which contexts. This lack of standardised guidance can create challenges for service providers and clinicians seeking to implement evidence‐based solutions.

Leading health organisations such as the WHO and the UK's National Institute for Health and Care Excellence (NICE) have both identified community‐based PSIs as a critical response to the growing challenge of social isolation in older adults (NICE 2015; WHO 2021). The WHO (2010) recognises isolation as a significant risk factor for mental decline in older age and recommends structured, community‐level interventions as both a preventive and therapeutic strategy. Similarly, NICE (2016) endorses befriending services, group activities and peer support schemes because of their potential to enhance social engagement and improve mental wellbeing. Governments across Ireland, the UK and the US have also begun prioritising social isolation in policy (HM Government 2018; U.S. Department of Health and Human Services 2023; Government of Ireland 2024). In Ireland, the *Healthy Age‐Friendly Homes* programme (Department of Health 2021) aims to help older people live independently whilst promoting community connection through housing and social support services. Ireland's *National Positive Ageing Strategy* (2013) also identifies social participation and community engagement as essential to promoting older adults' health, clearly addressing social exclusion and loneliness. The UK's Campaign to End Loneliness (2024) and the US *Surgeon General's Advisory on the Epidemic of Loneliness and Isolation* (2023) both call for wider adoption of psychosocial strategies to rebuild social connection at a population level (Department of Health and Human Services 2023; Campaign to End Loneliness 2024).

To date, although an increasing number of interventions targeting social isolation often interchange with loneliness, the effectiveness of PSIs implemented in the community with older adults experiencing social isolation has been overlooked. This review therefore aims to explore and evaluate the effectiveness of PSIs implemented in community settings to reduce social isolation in older adults and to synthesise the experiences of healthcare providers and older adults in response to PSIs. Specifically, the review seeks to answer the following research questions:

1. What are the experiences of older adults and healthcare professionals regarding PSIs within community settings?
2. Is there evidence that certain PSIs are more effective than others in reducing social isolation among this population?

## 3 Methods

### 3.1 Study Design

This integrative review is conducted with the rigour associated with a systematic review using Cochrane guidance (Higgins et al. 2024) and it is reported according to the Preferred Reporting Items for Systematic Reviews and Meta‐Analyses (PRISMA) guidelines (Page et al. 2021). An integrative review (Whittemore and Knafl 2005) was conducted as it allows the inclusion of empirical studies of any research design such as qualitative, quantitative or mixed methods and were eligible for inclusion provided they focused on PSIs for older adults to treat social isolation in the community. This review was guided by the five‐stage integrative review framework proposed by Whittemore and Knafl (2005) which includes: (1) problem identification, (2) literature search, (3) data evaluation, (4) data analysis and (5) presentation.

#### 3.1.1 Problem Identification

A preliminary search of the grey literature including the Open Grey database, ProQuest Dissertations and Theses Global, the Cochrane Library, Google Scholar and Prospero was conducted to ensure no other similar review had been conducted. This preliminary search found a dearth of literature related to PSIs for older adults to treat social isolation in the community. The Population, Exposure and Concept (PEC) framework guided the formulation of the research question and informed the development of search terms that aligned with the intended scope of the review. The PEC framework was used to structure the search strategy and refine the following review question: *What psychosocial interventions are being delivered to older adults in the community experiencing social isolation and how well do they work*?

### 3.2 Search Strategy

The search was conducted in December 2024 using five electronic databases: CINAHL, Medline, Web of Science, PsycINFO and Embase. To increase the overall relevance of the review, we restricted the search to those published between January 2013 and December 2024. No limit to language, geographic location or type of study was used to maximise retrieval. Search terms (File S1) using Medical Subject Headings (MeSH), thesaurus, associated free‐text terms and truncated terms were used to maximise retrieval and searched in all titles and abstracts and then combined using the Boolean operators ‘OR’ and ‘AND’. Reference lists of potentially eligible studies were all hand searched to avoid missing potentially eligible studies not retrieved by the database searches.

### 3.3 Eligibility Criteria

The PEC (Population, Exposure and Concept) framework was used to determine the following eligibility: (1) empirical studies that focused on older adults (≥ 65 years) experiencing social isolation; (2) focused on PSIs, including all non‐pharmacological interventions based on reducing social isolation; (3) were quantitative, qualitative, mixed‐methods or case studies research designs. Studies were excluded if they did not report on (1) the empirical studies outlined in the PEC framework; and (2) were reported from non‐peer‐reviewed theses, protocols, conference abstracts, editorials and letters to the editor to ensure the rigour of synthesis.

### 3.4 Study Selection

All retrieved citations from the databases were imported into Covidence (www.covidence.org) for removal of duplicates and systematic screening. Title and abstract screening was conducted independently by two reviewers (AK, LG), with full text review performed based on pre‐defined eligibility criteria. Any eligibility uncertainty or discrepancies were resolved through discussion and consensus among the review team.

### 3.5 Quality Assessment

Methodological quality and critical appraisal of the included studies were assessed independently by two authors (AK, AG) to ensure the rigour, credibility, accuracy and consistency of data. The quality of qualitative and quasi‐experimental studies was critically appraised using standard critical appraisal tools by the Joanna Briggs Institute (JBI) critical appraisal tools (JBI 2017) and the Revised Cochrane Risk of Bias Tool for Randomised Trials (ROB 2) was used to assess the methodological quality of Randomised Control Trials (RCTs) (see File S2).

### 3.6 Data Extraction

A Microsoft Excel data extraction form was developed to systematically extract the following key information from each study relevant to the research question: author(s), year, country, study aim(s)/objectives, study design, sample size, participants' characteristics, recruitment methods/eligibility criteria, data analysis, psychosocial intervention details using Template for Intervention Description and Replication (TIDieR) (Hoffmann et al. 2014), outcome measures, and key findings. This Microsoft Excel data extraction form was developed and piloted by the two authors (AK, AG) and the extraction process was performed independently by (AK) and cross‐checked for accuracy by (AG) to minimise reporting bias.

### 3.7 Data Analysis

Meta‐analysis was not plausible owing to the heterogeneity in study design, reported outcome measures and study outcomes. Instead, a narrative synthesis approach was employed following Popay et al.'s (2006) framework. Studies were grouped based on intervention type, study design and outcome measures. Patterns and themes were identified across studies to provide a comprehensive synthesis of the effectiveness of psychosocial interventions in reducing social isolation among older adults.

## 4 Results

**FIGURE 1:**
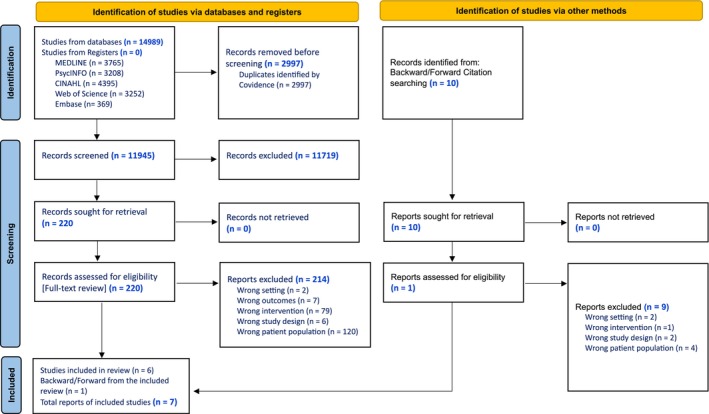
PRISMA 2020 flowchart of the study selection process.

A total of 14 989 records were retrieved from database searches, with 2997 duplicates removed. Following title and abstract screening, 220 full texts were assessed for eligibility, of which six (*n* = 6) studies met the inclusion criteria. An additional one (*n* = 1) study was identified through manual citation tracking bringing the final inclusion total to seven (*n* = 7) studies. Figure 1 provides an overview of the systematic search strategy and the process used to screen studies for eligibility.

### 4.1 Study and Participant's Characteristics

An overview of the study and participants' characteristics is presented in Table 1. Each study was undertaken between 2013 and 2023. The included studies reported a total of 464 participants experiencing social isolation. The studies were conducted across four (*n* = 4) countries/regions: three (*n* = 3) in Spain (Lapena et al. 2020; Santos‐Olmo et al. 2022; Hernández‐Ascanio et al. 2023), two (*n* = 2) in the United Kingdom (Hemingway 2013; Fakoya et al. 2023) and one (*n* = 1) each in the Republic of Ireland (Bantry‐White et al. 2018) and Canada (Lai et al. 2020). Four (*n* = 4) of the included studies were qualitative designs, using data from interviews and focus groups (Hemingway 2013; Bantry‐White et al. 2018; Lapena et al. 2020; Fakoya et al. 2023), two (*n* = 2) were RCTs (Lai et al. 2020; Hernández‐Ascanio et al. 2023) and one (*n* = 1) employed a quantitative design using a quasi‐experimental approach (Santos‐Olmo et al. 2022).

**TABLE 1:** Study and participant's characteristics.

Author(s), year, country	Study aim and design	Data collection/setting	Sample size and participants' characteristics	Data collection process	Data analysis	Findings
Hernández‐Ascanio et al. (2023) Spain	Aims: To assess the effect of a multicomponent intervention on reducing social isolation and loneliness and improving the quality of life in community‐dwelling older adults. Randomised Control Trial	Data were collected using the DUFFS for social isolation. The Jong‐ Gierveld Loneliness scale was used to measure loneliness. EuroQol‐ 5D (EQ‐ 5D) was used to assess HRQOL	The subjects participating in the study were recruited by consecutive sampling, through 32 healthcare professionals (3 general practitioner residents, 9 general practitioners and 20 nurses), from 13 healthcare centres.	The people in the intervention group were evaluated at three different time points: at the baseline, before the onset of the intervention (T1), a second‐ time point at the end of the intervention (4 months after the onset of the intervention) (T2), and a third final time point 2 months after the end of the intervention (T3). In the control group, only two measurements were performed, at baseline (T1) and 6 months after this measurement (T3).	Data analysed using inferential statistics, including t‐tests and ANOVA for between‐group comparisons, and regression analysis to assess intervention impact	The total DUFFS (social isolation) scores in the experimental group improved by almost three expected points (scores ranged from 25.00 to 27.94, *p* = 0.005) between the T1 and T2. An improvement in emotional loneliness scores between T2 and T3 was found (Wilcoxon test; *p* = 0.012) in the experimental group.
Lai et al. (2020) Canada	To examine the effectiveness of a peer‐based intervention in reducing loneliness, social isolation, and improving psychosocial well‐being with a sample of ageing Chinese immigrants. Randomised Control Trial	De Jong Loneliness Scale‐6. Lubben Social Network scale (LSNS). General Depression Scale (GDS‐4), the Geriatric Anxiety Inventory Short Form (GAI‐SF). Connor Davidson Resilience Scale (CD‐RISC 2). Ryff's Psychological Well‐being Scale. Life satisfaction was measured with a single question.	Sixty participants were recruited by promoting information about the CCHP in Chinese immigrant communities and independent living and assisted living facilities for Chinese older people in the community.	Twenty‐four volunteers aged 48 to 76 engaged in two‐on‐one peer support through home visits and telephone calls to provide emotional support, problem‐solving support, and community resource sharing. Social workers who are not blinded to the group assignment measured the changes of both the intervention group and the control group participants in a range of psychosocial outcomes including three primary outcomes (loneliness, social support, barriers to social participation) and five secondary outcomes (depressive symptoms, anxiety, life satisfaction, happiness, and purpose in life).	Multivariate regression analyses	The intervention group participants reported a mean decrease of 1.17 units in loneliness (95% CI, 0.45 to 1.89), a mean decrease of 0.57 units in barriers to social participation (95% CI, 0.13 to 1.00), and a mean increase of 1.37 units in resilience (95% CI, 0.70 to 2.03). Intervention group participants also experienced significantly increased levels of happiness, a smaller per centage of participants reporting depressive symptoms, and a higher percentage of participants reporting life satisfaction.
Santos‐Olmo et al. (2022) Spain	To describe the components of the Psychological Support Service for Socially Isolated Elderly People (PSIE) and analyse its effectiveness in Madrid. Quasi‐experimental	Home‐based assessments; direct observations, interviews with users and networks.	Referred by Madrid City Council social services.	HoNOS65+, GAF, WHO‐DAS‐S, CANE. Home Health Outcome Scales for People Over 65 (HoNOS65+) in its Spanish adaptation. Global Assessment of Functioning (GAF) in its Spanish adaptation. WHO Brief Disability Assessment Scale (WHO‐DAS‐S) [27] in its Spanish adaptation. Camberwell Needs Assessment Questionnaire for the Elderly (CANE) in its Spanish adaptation.	Chi‐squared tests, Student's t‐tests, repeated measures ANOVA, Cohen's d for effect size.	Health and Psychosocial Functioning (HoNOS65+): Significant improvements in behavioural disturbance, alcohol use, psychotic symptoms, social relationships, and living conditions. Total score reduced significantly from 21.5 (pre‐intervention) to 13.72 (post‐intervention), indicating better functioning. Global Functioning (GAF): Increased mean scores from 44.67 to 52.24, reflecting better global functioning (medium effect size, *d* = 0.6). Disability (WHO‐DAS‐S): Improved scores across personal care, occupational, family, and leisure domains, with large effect sizes (*d* = 0.93–1.3). Social Health Needs (CANE): Reduction in unmet needs from 10.68 (pre‐intervention) to 1.44 (post‐intervention), with a large effect size (*d* = 2.31).
Bantry‐White et al. (2018) Ireland	The focus of this study was on the sociocultural context of intervention, examining the underlying understandings of social isolation and loneliness within a rural community, and the implications of these for intervention design, context‐sensitivity and acceptability. Qualitative study	Semistructured in‐depth interviews were carried out with eight participants to facilitate participant‐led responses broadly on the themes of social interaction and connectedness, loneliness and their experience of befriending. These were expanded upon as themes emerged at interview. The interview was generative and aimed to reflect sociocultural understandings of rural community in Ireland that hold relevance beyond the research site. Two focus groups, comprised of 10 befrienders, were also conducted, with each lasting circa 60 min.	Participants in receipt of befriending included six women and two men, aged 58–92 years (median 76.88 years). Users of the service were primarily older people but included some younger people identified as isolated on the basis of a health issue. Efforts were made to select participants that would elicit a diversity of perspectives; sample selection aimed to include women and men, people who lived alone and those who had lived away from the area for a period of time. Participants were identified purposively through the service's co‐ordinators	Semistructured in depth interviews and focus groups.	All data were audio‐recorded, transcribed and analysed through QSR NViVo 10 software. The analytical framework of the study was an ‘interpretive one in search of meaning of people's conceptual worlds’. It involved interpreting the ‘websof significance’ (spun) by the befrienders, the befriended and the community workers across two themes: (i) the symbolic construction of social isolation and loneliness through the lens of community and (ii) the representation of community within the befriending intervention. The data were independently analysed by two researchers in stage one and the analysis was collaboratively combined by the research team and consensus on findings reached in stage two.	Highlighted the importance of neighbourhood‐level interventions and informal community support networks in reducing isolation.
Fakoya et al. (2023) United Kingdom	This study aimed to elicit from the perspective of a range of service providers, the barriers and facilitators of successful service provision, and the reduction of loneliness among older people. Qualitative study	Semistructured interviews focusing on barriers and facilitators to service success.	A range of methods was used to identify eligible services, including Internet trawls, contacting key stakeholders known to the research team, and snowball sampling.	Semistructured interviews.	Thematic analysis following Braun and Clarke's framework. Coding conducted iteratively, with themes reviewed by co‐authors.	Participants appreciated the individualised attention and support received. Reported increased feelings of inclusion and connection to their communities. Healthcare providers and volunteers the work rewarding but resource intensive. Emphasised the need for proper training and consistent funding to sustain services.
Lapena et al. (2020) Spain	The aim of this study was to explore participants' and organisers' perceptions of the implementation process of the ‘School of Health’, by identifying barriers and facilitators to participation and positive and negative aspects of the programme. This study also aimed to describe the perceived benefits of the community intervention on loneliness and social isolation among participants and to elicit proposals to improve the intervention. Qualitative study	Four focus groups (two per neighbourhood, stratified by loneliness risk). Two semistructured interviews with coordinators. Session's audio‐recorded and transcribed verbatim.	Referrals from primary care, social services, and community centres; posters and direct invitations.	Semistructured interviews	All recordings were transcribed with verbatim and data that identified informants were anonymised. A thematic content analysis was carried out with the support of Atlas.ti software (ATLAS.ti version: WIN 7.5 build 12). Preanalytical intuitions were formulated after successive readings of the transcriptions and the observation notes.	Participants noted improved relationships and opportunities to meet new people within their neighbourhood. Reconnection with acquaintances and peers enhanced a sense of community. The program encouraged participants to break habits of isolation and provided structure to their weeks. Participation fostered feelings of being part of a group or community.
Hemingway (2013) United Kingdom	To explore and describe the phenomenon of social isolation from the perspective of older adults. Qualitative study	Participant observation. Individual interviews. Focus groups. Biographical information collection from club members.	Facilitated by club leaders and supported by word‐of‐mouth among members.	Qualitative measures to capture participant insights. Interviews and focus groups recorded, transcribed and analysed. Reflexive journaling and field notes kept by researchers.	Modified inductive content analysis and constant comparative method.	Enhanced friendships and mutual emotional support. Peer interactions improved confidence and reduced isolation.

The sample sizes across the studies ranged from 22 to 121 participants, 287 (61.9%) identified as female and 171 (38.1%) as male with ages ranging from 58 to 90 years. All studies recruited participants aged over 65 years but Bantry‐White et al. (2018) recruited one participant aged 58. Despite this, the study was included because it provided valuable insights into the role of neighbourhood‐level interventions and informal community networks in reducing social isolation among both older adults and those nearing older age. Ethnicity was reported in five (*n* = 5) studies (Hemingway 2013; Bantry‐White et al. 2018; Lai et al. 2020; Santos‐Olmo et al. 2022; Hernández‐Ascanio et al. 2023) where a majority identified as Caucasian (*n* = 345, 74.4%), White British (*n* = 65, 14%), Asian (*n* = 60, 12.9%), indigenous or minority ethnic groups (*n* = 14, 3%), other (*n* = 5, 1.1%), and an additional 40 participants (8.6%) were unspecified.

### 4.2 Methodological Quality Assessment

Quality appraisal was conducted on all included studies not with a view to excluding the study from the review but to identify the quality of the available evidence. Five (*n* = 5) studies demonstrated good methodological quality based on the JBI Critical Appraisal Checklists (File S2). A total of four (*n* = 4) qualitative studies (Hemingway 2013; Bantry‐White et al. 2018; Lapena et al. 2020; Fakoya et al. 2023) did not report the researchers' cultural or theoretical positions, nor did they reflect on the potential influence of the researcher. The two (*n* = 2) RCTs (Lai et al. 2020; Hernández‐Ascanio et al. 2023) assessed using the revised Cochrane ROB2 were classified as having a ‘high/low risk of bias’, primarily due to limitations in outcome measurement. None of the studies were excluded based on their methodological assessment. The rigorous appraisal process adopted in this review strengthens the credibility and robustness of the findings.

### 4.3 Characteristics and Components of Psychosocial Interventions

The characteristics of the included PSIs were adapted from the Template for Intervention Description and Replication (TIDieR) checklist (Hoffmann et al. 2014). The selected PSIs used in the included studies focused on various approaches to mitigate social isolation among older adults. These interventions align with key frameworks, including community‐based programs, cognitive‐behavioural strategies, social support mechanisms and digital solutions, ensuring a structured and evidence‐based approach to addressing social isolation. PSIs often incorporated wellness programs focusing on mental health, physical activities and lifestyle modifications to enhance overall well‐being. Guided relaxation techniques, physical activity programs, and nutritional guidance were included in interventions to address both physical and emotional aspects of social isolation (Hemingway 2013; Santos‐Olmo et al. 2022).

Community‐based interventions used group activities and social engagement to promote a sense of belonging (Bantry‐White et al. 2018; Lai et al. 2020). Social engagement and peer support were found to be crucial as group activities, including structured social gatherings, arts‐based interventions and peer‐led discussion groups helped foster social connection and reduce social isolation (Bantry‐White et al. 2018; Lai et al. 2020). Community‐based interventions reinforced a sense of belonging and encouraged sustained social interactions, which were essential for maintaining long‐term social well‐being (Lapena et al. 2020).

Cognitive behavioural interventions employed techniques such as cognitive restructuring and emotional regulation to modify negative thought patterns associated with social isolation including social relationships and emotional support (Hemingway 2013; Santos‐Olmo et al. 2022). Cognitive behavioural strategies played an important role, particularly through emotional support mechanisms such as cognitive reframing, mindfulness training and resilience‐building exercises. These interventions incorporated structured activities such as guided discussions and reflective exercises to assist participants in managing distressing emotions and enhancing coping mechanisms (Hemingway 2013; Santos‐Olmo et al. 2022).

Whilst not an intervention but a common goal, several of the studies discussed person‐centred approaches, which the review team deemed important to the overall effectiveness of a PSI being effective in reducing social isolation. Person‐centred care approaches focused on meaningful engagement by tailoring interventions to individual needs and cultural contexts (Lapena et al. 2020). Person‐centred care and customisation were also essential, as interventions tailored to individual preferences and cultural backgrounds proved to be more effective in nurturing meaningful connections (Lapena et al. 2020). Personalised approaches included mentorship programs, buddy systems and individualised activity plans aligned with participants' interests and social needs.

### 4.4 The Effects and Impact of PSI on Social Isolation

The impacts of PSIs were assessed using different study designs (Table 2). Two RCTs (Lai et al. 2020; Hernández‐Ascanio et al. 2023) demonstrated significant improvements in social connectivity and mental well‐being. Hernández‐Ascanio et al. (2023) examined a multicomponent intervention incorporating home‐based sessions, reminiscence therapy, goal setting and communication skills training. This study found a significant improvement in total social isolation scores within the experimental group, an effect that was maintained and continued to improve two months after the intervention ended. The findings also demonstrated enhanced participant ability to sustain social connections through direct engagement strategies. Lai et al. (2020) investigated a peer‐based intervention targeting older Chinese immigrants and reported statistically significant reductions in social isolation. The peer‐based intervention was also effective in enhancing resilience and life satisfaction among socially isolated older adults, particularly within ethnic minority immigrant populations. In a quasi‐experimental study, Santos‐Olmo et al. (2022) examined the impact of the structured Psychological Support Service for Socially Isolated Elderly People (PSIE), an intervention designed to reduce social isolation among older adults. PSIE was found to strengthen participants' social networks, facilitate acceptance of various social resources, and connect socially isolated individuals with regular social and health services. It also helped shift participants' attitudes by encouraging them to accept previously declined support, engage with community members (such as neighbours and shopkeepers), and re‐establish contact and relationships with family members.

**TABLE 2:** Effects of psychosocial interventions on social isolation.

Types of interventions	Study	Outcomes	Effects
Modified CARELINK Program	Hernández‐Ascanio et al. (2023)	Duke‐UNC Functional Social Support Questionnaire (DUFSS). Loneliness and quality of life	The total DUFFS (social isolation) scores in the experimental group improved by almost three expected points (scores ranged from 25.00 to 27.94, *p* = 0.005) between the T1 and T2. In addition, the effect of the intervention on this variable was maintained and still improved 2 months after the end of the intervention, that is, at T3 (reaching 28.5, *p* = 0.002 vs. T1)
Chinese Community Helpers Program (CCHP)—Peer‐based intervention on loneliness and social isolation	Lai et al. (2020)	Loneliness, Life Satisfaction, Social Support, Depression, Anxiety, Resilience.	Participants felt less isolated and more integrated. Improved Resilience—Support enhanced participants' ability to adapt to challenges
Psychological Support Service for Socially Isolated Elderly People (PSIE)	Santos‐Olmo et al. (2022)	Home Health Outcome Scales for People Over 65, Global Assessment of Functioning (GAF), WHO Brief Disability Assessment Scale (WHO‐DAS‐S). Camberwell Needs Assessment Questionnaire for the Elderly (CANE).	Significant improvements in behavioural disturbance, alcohol use, psychotic symptoms, social relationships, and living conditions. Increased mean scores from 44.67 to 52.24, reflecting better global functioning (medium effect size, *d* = 0.6). Disability (WHO‐DAS‐S)—Improved scores across personal care, occupational, family, and leisure domains, with large effect sizes (*d* = 0.93–1.3). Social Health Needs (CANE)—Reduction in unmet needs from 10.68 (pre‐intervention) to 1.44 (post‐intervention), with a large effect size (*d* = 2.31).
Rural Befriending Programme	Bantry‐White et al. (2018)	Qualitative exploration of community, social isolation, and loneliness.	Participants described deep attachments to their rural locality, emphasising shared histories and geographical ties. Perceived loss of community landmarks, such as churches and community halls, highlighted the erosion of traditional gathering spaces. A sense of belonging was fostered through shared histories, activities, and cultural norms. Gendered activities reinforced bonds but limited inclusivity (e.g., men preferred historically significant activities, whilst women favoured social or crafting events). Informal networks and reciprocity (e.g., neighbourly support) were central to the community's social fabric. Befriending relationships often mirrored traditional community values but were seen as a substitute for organic connections.
Interventions categorised by the Campaign to End Loneliness (CTEL) framework	Fakoya et al. (2023)	Service effectiveness discussed in terms of thematic outcomes (e.g., engagement, accessibility, user satisfaction).	Encouraging autonomy among participants and staff builds confidence and satisfaction. Participation in the design and delivery of interventions creates a sense of ownership. Personal Qualities of Staff and Volunteers—Empathy, caring nature, and local knowledge strengthen rapport and trust. Willingness to ‘*go above and beyond*’ by volunteers creates valued relationships. Funding Challenges—Inconsistent funding limits long‐term planning and service reach. Stigma and mobility issues hinder service access for vulnerable populations. Enhancing awareness of loneliness symptoms and available services can normalise seeking help.
School of Health for Older People	Lapena et al. (2020)	The aim of this study was to explore participants' and organisers' perceptions of the implementation process of the ‘School of Health’, by identifying barriers and facilitators to participation and positive and negative aspects of the programme.	Participants noted improved relationships and opportunities to meet new people within their neighbourhood. Reconnection with acquaintances and peers enhanced a sense of community. Participation fostered feelings of being part of a group or community. Mobility issues and other health problems hindered attendance. Emotional challenges, such as a lack of motivation or depression, were also cited. Family Obligations—Caregiving responsibilities for grandchildren or spouses limited availability.
Friendship Clubs	Hemingway (2013)	Qualitative outcomes included participants' perceptions of social connectedness, emotional well‐being, and barriers to participation.	Social Relationships—Enhanced friendships and mutual emotional support. Peer interactions improved confidence and reduced isolation. Barriers—Mobility challenges and financial constraints limited attendance. Fear of public spaces or stigma around loneliness. Empowerment—Attendees and volunteers viewed themselves as assets, creating a sense of purpose and value.

Four qualitative studies (Hemingway 2013; Bantry‐White et al. 2018; Lapena et al. 2020; Fakoya et al. 2023) also provided insights into the experiences and perceptions of PSIs for older adults. Findings indicated enhanced quality of life, with interviews and focus groups suggesting that person‐centered approaches increased satisfaction and overall well‐being (Hemingway 2013; Lapena et al. 2020). Participants described increased feelings of belonging and emotional well‐being as key benefits of these PSIs (Bantry‐White et al. 2018; Fakoya et al. 2023).

#### 4.4.1 Outcome Measures

Various outcome measures were employed to evaluate changes in social isolation and psychosocial well‐being. The Duke Social Support Index (DUFSS) (Bellón et al. 1996) showed a significant impact on social connectedness (Bantry‐White et al. 2018; Santos‐Olmo et al. 2022), reporting a 15%–25% increase in social support perceptions. The Quality of Life (QoL) (Herdman et al. 2011) scale indicated enhancements in emotional well‐being, daily functioning and overall life satisfaction post intervention (Hemingway 2013; Lapena et al. 2020). The Lubben Social Network Scale (LSNS) (Lubben et al. 2006) reported significantly higher scores indicating that interventions emphasising peer support and structured social activities led to increased social connections (Hernández‐Ascanio et al. 2023; Fakoya et al. 2023). The De Jong Gierveld Loneliness Scale (De Jong Gierveld and Van Tilburg 2006) showed significant reductions in perceived social loneliness and social isolation, particularly in interventions incorporating cognitive behavioural techniques (Lai et al. 2020). The Hospital Anxiety and Depression Scale (HADS) (Zigmond and Snaith 1983) was used in several studies, indicating that PSIs not only reduced social isolation but also led to decreases in mental health symptoms, particularly anxiety and depression, with reported reductions in symptom scores ranging from 25% to 40% (Hemingway 2013; Santos‐Olmo et al. 2022).

#### 4.4.2 Comparison of Group‐ and Individual‐Based PSIs

The effectiveness of PSIs in reducing social isolation, depression and anxiety varied depending on whether they were delivered in a group‐based or individual‐based format. Group‐based interventions generally strengthen social connection and peer support, whilst individual‐based interventions often provide more tailored psychological support. Group‐based interventions, such as peer‐led discussion groups, social gatherings and structured community activities, were particularly effective in reducing social isolation. Bantry‐White et al. (2018) and Lai et al. (2020) demonstrated that older adults participating in community‐based social programs reported significant improvements in social connectedness and feelings of belonging. These interventions create shared experiences, reduce the stigma associated with social isolation and promote sustained social participation. However, the impact of group‐based interventions on anxiety and depression was mixed. Although some participants benefitted from the emotional support of peers, others found it difficult to engage due to social anxiety or discomfort in group settings (Hernández‐Ascanio et al. 2023). Additionally, some individuals were hesitant to participate, particularly in interventions that required active engagement or emotional disclosure (Fakoya et al. 2023).

In contrast, individual‐based interventions, such as counselling, cognitive behavioural therapy (CBT) and reminiscence therapy, demonstrated stronger effects on anxiety and depression. Cognitive behavioural approaches were particularly beneficial in helping participants reframe negative thoughts, develop coping mechanisms and enhance emotional resilience. Studies show that CBT interventions led to a 25%–35% reduction in anxiety and depressive symptoms (Hemingway 2013; Santos‐Olmo et al. 2022). Individual interventions allowed for personalised support and were particularly effective for individuals who preferred privacy or had difficulty engaging in group settings.

Despite their effectiveness in reducing anxiety and depression, individual interventions were less effective in improving social connection. Participants who received one‐to‐one therapy benefitted from psychological support, but unless additional social engagement components were incorporated, they did not experience the same degree of improvement in social relationships as those in group‐based interventions (Lapena et al. 2020). Some studies suggest that a hybrid model, combining group‐based social support with individual psychological counselling may offer the most comprehensive benefits (Hernández‐Ascanio et al. 2023).

#### 4.4.3 Healthcare Professionals/Caregivers Perspective of Delivering PSIs

The experiences of healthcare professionals and caregivers delivering PSIs varied. Some found the interventions rewarding as they observed improvements in participants' emotional well‐being and social connectedness (Hernández‐Ascanio et al. 2023; Fakoya et al. 2023). However, others reported challenges related to high workloads, lack of adequate training and emotional burden when working with individuals experiencing social isolation (Lapena et al. 2020; Fakoya et al. 2023). Many professionals noted increased job satisfaction from witnessing tangible improvements in participants' well‐being. One caregiver stated, ‘It's incredibly rewarding to see someone regain their confidence and connect with others after months of isolation’ (Fakoya et al. 2023).

Some professionals expressed concerns about emotional fatigue, with one healthcare worker stating, ‘Providing emotional support can be draining. More training and structured debriefing sessions would help us sustain our roles’ (Santos‐Olmo et al. 2022). Others remarked on difficulties engaging participants, especially those initially reluctant to participate. One professional shared, ‘Some participants were reluctant at first, but with time and consistent encouragement, they started engaging more’ (Hernández‐Ascanio et al. 2023).

It is also important to acknowledge what did not work as well. Some studies highlighted that interventions lacking consistency, clear structure, or cultural relevance tended to have limited impact (Bantry‐White et al. 2018; Santos‐Olmo et al. 2022). For instance, when sessions were too short or infrequent, participants found it difficult to build relationships or engage meaningfully (Lapena et al. 2020). In a few cases, group interventions failed to reduce isolation when participants were reluctant to ‘open up’ in front of others, highlighting the importance of matching intervention types to personal preferences and needs (Fakoya et al. 2023).

#### 4.4.4 Participant's Perspective of Receiving PSIs

Qualitative studies (Hemingway 2013; Lapena et al. 2020) included in this review revealed positive experiences among older adults participating in PSIs and reported positive experiences, highlighting improvements in social connectedness, mental well‐being and self‐esteem. Many participants expressed feeling less isolated and valued the opportunity to form meaningful relationships. One participant shared, ‘I felt invisible before, but now I have people who care about me and check in regularly’ (Lapena et al. 2020). Others noted reduced anxiety and depression symptoms, particularly in cognitive behavioural and peer support interventions. One participant stated, ‘The support group gave me a space to talk about my feelings without being judged. It helped me feel lighter’ (Hemingway 2013). Participants appreciated both the social interaction and the emotional support provided by structured interventions, stating that they felt ‘*seen*’ and ‘*valued*’ for the first time in years. Many older adults talked about feeling more visible, valued and emotionally lighter after engaging in an intervention. These voices make it clear that the benefit of these interventions can be life changing.

## 5 Discussion

This integrative review explored the effectiveness of PSIs implemented in community settings for reducing social isolation among older adults. Based on both qualitative and quantitative evidence, the findings demonstrate that whilst PSIs can significantly improve social connectedness, reduce social isolation and increase mental well‐being, their effectiveness is highly dependent on intervention design, delivery format, individual needs and context. This review found that PSIs increased perceptions of social support, rising by 15%–25% in some studies, whilst reducing symptoms of anxiety and depression. These outcomes suggest that PSIs when tailored to the needs of older adults, can produce changes in both social and emotional domains. These findings are supported by a recent study by Min et al. (2023) which demonstrated that targeted interventions reduce hospitalisation and emergency visits. Similarly, the Substance Abuse and Mental Health Services Administration (SAMHSA) (2021) emphasised the importance of early, person‐centred approaches to improve outcomes in older adults with complex mental health needs. Such personalised approaches are also recommended by policy and guidelines, including those published by NICE (2016), which recommend specific interventions, such as walking schemes and singing programmes that consider individual needs to effectively address social isolation and improve mental well‐being in older populations. Another intervention recommended by NICE (2016) is those that are intergenerational in nature; an example of which is provided by Tuohy et al. (2023) who identified that intergenerational cafés could provide participation and social connection for older adults whilst also increasing a sense of understanding of the experiences of older adults.

Participants recruited in the qualitative studies (Hemingway 2013; Lapena et al. 2020) who received PSIs generally reported positive experiences, highlighting improvements in social connectedness, mental well‐being and self‐esteem. These findings are consistent with those of Courtin and Knapp (2017), who emphasised the importance of meaningful social relationships in enhancing psychological health and quality of life among older adults. The findings from this review provide important insights into which strategies work, for whom and under what circumstances and add to the growing literature on addressing social isolation in aging populations. Although many staff found PSIs meaningful and rewarding, issues such as high workloads, lack of training and emotional fatigue were common (Lapena et al. 2020; Santos‐Olmo et al. 2022; Fakoya et al. 2023). Structured support for staff, including training and supervision (Rapaport et al. 2023), may therefore be essential to maintain program quality and sustainability in PSIs for older adults (Hunter et al. 2016; Nagel et al. 2023; SAMHSA 2021). Overall, this review suggests that combining group and individual approaches is likely to offer the most benefit. PSIs that are flexible, person‐centered, and culturally inclusive appear to work best. Programs that offer peer connection combined with psychological support are particularly beneficial (Hernández‐Ascanio et al. 2023; Horgan et al. 2024). This review also aligns with recommendations made by WHO (2012) and NICE (2015) regarding the use of structured, community‐based psychosocial approaches to support older adults.

Group‐based interventions were very effective in helping people to reconnect socially. Programmes that brought older adults together (Bantry‐White et al. 2018; Lai et al. 2020) whether through arts, discussion groups, or peer‐led activities, helped participants feel more connected, supported and part of a community. This also helped them to feel less alone, more resilient after taking part in a peer support programme and more confident about socialising. The shared experience of being with others in a similar situation helped people feel understood and less isolated. This is consistent with findings from Gardiner et al. (2018), who noted that social interventions are more effective when they promote ongoing interaction and shared purpose.

The impact of group‐based PSIs on mental health outcomes such as anxiety and depression varied. Some participants found group settings a challenge due to social anxiety or discomfort, which limited their ability to fully engage (Fakoya et al. 2023). This supports the argument that flexibility in delivery can be crucial for accessibility (Noone et al. 2020). Individual interventions such as counselling, CBT, or reminiscence therapy were particularly useful for helping people who might be experiencing low mood, anxiety or difficulty ‘opening up’ in a group. These approaches were especially effective in helping people manage the emotional impact of isolation and build personal coping skills. These findings were similar to studies by Zhang et al. (2023) and Horgan et al. (2024), which found that PSIs are effective in reducing symptoms of depression and anxiety among older adults. It was noted that CBT helped reduce anxiety and depression symptoms by as much as 25%–35%. It is important to note that whilst individualised interventions were beneficial for psychological well‐being, they were generally less effective in improving social networks unless combined with group or community engagement components (Lapena et al. 2020). These findings are echoed by Fakoya et al. (2020) and Windle et al. (2011), who highlight the complementary nature of group and individual interventions. These results underscore why PSIs should be an essential part of care for older adults. By targeting the emotional and social dimensions of isolation, PSIs offer a comprehensive approach that can improve mental health, enhance quality of life and reduce the long‐term burden on the healthcare system.

### 5.1 Strengths and Limitations

One notable strength of this review is its clear distinction between social isolation and loneliness. Although the two are often discussed interchangeably, this review focused specifically on social isolation as an objective lack of social contact, rather than the subjective feeling of loneliness. This allowed for a more precise exploration of how PSIs target different aspects of social isolation. Also, this review integrates evidence from both quantitative and qualitative studies, offering a more holistic understanding of intervention impact. By including the perspectives of older adults as well as healthcare professionals delivering interventions, it captures not just what works, but why it works or does not work in practice. This dual focus adds depth to the findings and supports the development of practical, person‐centred approaches to addressing isolation.

Although this review presents several strengths, it is also important to acknowledge its limitations. First, only seven (*n* = 7) studies met the inclusion criteria, which may limit the breadth, generalisability and transferability of the findings. Second, the included studies varied widely in terms of intervention type, duration and outcome measures, making direct comparisons and synthesis more challenging. This heterogeneity also limited the ability to determine which intervention components are most consistently effective across different populations and settings. Another limitation relates to the limited availability of long‐term follow‐up data. Most studies reported short‐term outcomes, and it remains unclear whether the benefits of PSIs are sustained over time.

## 6 Conclusion

This review demonstrates that PSIs, when tailored to the needs of older adults, can significantly reduce social isolation and enhance well‐being. Group‐based approaches helped promote a sense of connection and belonging, whilst individualised interventions supported emotional resilience and personal growth. The evidence suggests that interventions offering emotional support alongside opportunities to connect socially tend to be the most impactful. Importantly, the review offered a balanced view of both the benefits and challenges of delivering these interventions, highlighting the need for well‐structured, inclusive and sustainable models of care.

## 7 Relevance for Clinical Practice

The findings of this review highlight several important implications for healthcare policy and practice. First, the consistent positive outcomes associated with both group‐based and individual psychosocial interventions suggest that such programmes should be more widely integrated into community and primary care services for older adults. Policies (NICE 2015; WHO 2021; SAMHSA 2021) and studies (Gardiner et al. 2018; Hoang et al. 2022; Nagel et al. 2023) all advocate the support for PSIs and the delivery of structured person‐centred interventions to help address the growing public health challenge of social isolation in ageing populations. The review also highlights the importance of training and support for healthcare professionals involved in delivering these interventions (Hunter et al. 2016; Nagel et al. 2023). Ensuring that staff are equipped with the skills and resources needed to manage the emotional demands of this work would be crucial to sustaining effective service delivery (Rapaport et al. 2023; SAMHSA 2021).

This review highlights several areas for future research. First, more large‐scale high‐quality studies are needed to assess the long‐term effectiveness of PSIs for reducing social isolation in older adults. Many existing studies (Santos‐Olmo et al. 2022; Lapena et al. 2020) had small sample sizes and short follow‐up periods, limiting the ability to draw strong conclusions about sustained impact. Future studies should also explore which specific components of interventions, such as frequency, duration and mode of delivery, are most effective across different contexts and populations. Further investigations into the experiences of both participants and providers of interventions would be valuable in designing programmes that are not only effective but also feasible and acceptable in community‐based settings.

## Author Contributions

**Ann‐Marie Keane:** conceptualisation, formal analysis, investigation, data curation, writing – original draft preparation, writing – reviewing and editing, visualisation. **Ann‐Marie Bright:** formal analysis, validation, supervision, writing – reviewing and editing. **Lucan Greenan:** formal analysis, investigation, writing – reviewing and editing. **Annmarie Grealish:** conceptualisation, formal analysis, investigation, validation, supervision, writing – reviewing and editing.

## Funding

The authors have nothing to report.

## Ethics Statement

The authors have nothing to report.

## Conflicts of Interest

The authors declare no conflicts of interest.

## Supporting information


**File S1:** Full search strategy.

**File S2:** Quality assessment.

## Data Availability

The data that supports the findings of this study is available in the Supporting Information of this article.
