# Supplementary material for: The Impact of Psychosocial Interventions on Older Adults in the Community Experiencing Social Isolation: An Integrative Review
Source: Int J Ment Health Nurs. 2025 Dec 2;34(6):e70186. doi: 10.1111/inm.70186 (PMC12673195; doi:10.1111/inm.70186)
Supplement: Supplementary file 1 — File S1: Full search strategy. [file INM-34-0-s001.docx]

**Supplementary File 1: Full Search Strategy**

|  | ***Search done on PsycINFO, Medline, CINAHL*** | | ***PsycINFO*** | ***Medline*** | ***CINAHL*** |
| --- | --- | --- | --- | --- | --- |
|  | ***PsycINFO***  **Limiters** - Publication Year: 2014-2024; Publication Date: 20140101-20241131; Peer Reviewed; Publication Type: Peer Reviewed Journal; Age Groups: Aged (65 yrs & older); Population Group: Human; Document Type: Journal Article; Exclude Dissertations  **Expanders** - Apply equivalent subjects  **Narrow by SubjectMajor:**- psychosocial factors  **Narrow by SubjectMajor:**- social networks  **Narrow by SubjectMajor:**- social interaction  **Narrow by SubjectMajor:**- social isolation  **Narrow by SubjectMajor:**- loneliness  **Narrow by SubjectMajor:**- social support  **Narrow by SubjectMajor:**- older adulthood  **Narrow by SubjectMajor:**- aging  **Search modes** - Proximity | ***Medline***  Limiters - Publication Date: 20140101-20241131; Abstract Available; Human; Age Related: Aged: 65+ years; Publication Type: Journal Article; Peer Reviewed  Expanders - Apply equivalent subjects  Narrow by SubjectMajor: - stress, psychological  Narrow by SubjectMajor: - social participation  Narrow by SubjectMajor: - mental health  Narrow by SubjectMajor: - social isolation  Narrow by SubjectMajor: - loneliness  Narrow by SubjectMajor: - aging  Narrow by SubjectMajor: - social support  Narrow by SubjectMajor: - quality of life  Search modes – Proximity | **3,208** | **3,765** | **4,395** |
|  | **Medline, CINAHL** S1 and S2 and S3  Limiters - Publication Date: 20140101-20241131; Abstract Available; Human; Age Related: Aged: 65+ years; Publication Type: Journal Article; Peer Reviewed  Expanders - Apply equivalent subjects  Search modes - Proximity | | **----** | **13,625** | **4,395** |
| S5 | S1 AND S2 AND S3  Limiters - Publication Year: 2014-2024; Publication Date: 20140101-20241131; Peer Reviewed; Publication Type: Peer Reviewed Journal; Age Groups: Aged (65 yrs & older); Population Group: Human; Document Type: Journal Article; Exclude Dissertations | | **4,955** | **13,625** | **4,395** |
| S4 | S1 AND S2 AND S3 | | **33,595** | **56,790** | **24,270** |
| S3 | TI ( social isolation OR social exclusion* OR social alienation* OR social* isolat* OR social connect* OR *social support OR social participat* OR social relation* OR ostracism OR loneliness OR lonel* OR alone OR interpersonal relations OR social interaction OR social adjustment OR solitude OR solitary OR aloneness OR social participation ) OR AB( social isolation OR social exclusion* OR social alienation* OR social* isolat* OR social connect* OR *social support OR social participat* OR social relation* OR ostracism OR loneliness OR lonel* OR alone OR interpersonal relations OR social interaction OR social adjustment OR solitude OR solitary OR aloneness OR social participation ) | | **333,384** | **915,606** | **208,029** |
| S2 | TI ( psychosocial intervention OR Psychosocial OR Psychotherapy OR Social Support OR psychological OR psychoeducation* OR psychotherapy* OR psychotherapeutic OR intervention OR treatment* OR therap* OR behavioural OR behavioral OR counsel* ) OR AB ( psychosocial intervention OR Psychosocial OR Psychotherapy OR Social Support OR psychological OR psychoeducation* OR psychotherapy* OR psychotherapeutic OR intervention OR treatment* OR therap* OR behavioural OR behavioral OR counsel* ) | | **1,993,659** | **9,284,839** | **2,108,866** |
| S1 | TI ( aged OR elder* Or old* OR geriatric* OR aging OR ageing OR senior* OR older adults OR older* OR elder* OR senior* OR geriatric OR late life OR old age OR elder$ ) OR AB ( aged OR elder* Or old* OR geriatric* OR aging OR ageing OR senior* OR older adults OR older* OR elder* OR senior* OR geriatric OR late life OR old age OR elder$ ) | | **698,840** | **2,176,141** | **694,357** |

**Embase and Web of Science**

|  | **Search done on Embase and Web of Science** | **Embase** | **Web of Science** |
| --- | --- | --- | --- |
|  | **Web of Science** #1 AND #2 AND #3 on  Timespan: 2014-01-01 to 2024-11-30 (Publication Date) | **369** | **3,252** |
| **#4** | #1 AND #2 AND #3 | **369** | **4,637** |
| **#3** | 'social isolation':ti,ab OR 'social exclusion':ti,ab OR 'social alienation':ti,ab OR 'social isolat':ti,ab OR 'social connect':ti,ab OR 'social support':ti,ab OR 'social participat':ti,ab OR 'social relation':ti,ab OR ostracism:ti,ab OR loneliness:ti,ab OR lonel:ti,ab OR alone:ti,ab OR 'interpersonal relations':ti,ab OR 'social interaction':ti,ab OR 'social adjustment':ti,ab OR solitude:ti,ab OR solitary:ti,ab OR aloneness:ti,ab OR 'social participation':ti,ab | **1,123,955** | **1,603,467** |
| **#2** | (ti:ab,ti AND ('psychosocial intervention':ab,ti OR psychosocial:ab,ti OR psychotherapy:ab,ti OR 'social support':ab,ti OR psychological:ab,ti OR psychoeducation*:ab,ti OR psychotherapy*:ab,ti OR psychotherapeutic:ab,ti OR intervention:ab,ti OR treatment*:ab,ti OR therap*:ab,ti OR behavioural:ab,ti OR behavioral:ab,ti OR counsel*:ab,ti) OR ab:ab,ti) AND ('psychosocial intervention':ab,ti OR psychosocial:ab,ti OR psychotherapy:ab,ti OR 'social support':ab,ti OR psychological:ab,ti OR psychoeducation*:ab,ti OR psychotherapy*:ab,ti OR psychotherapeutic:ab,ti OR intervention:ab,ti OR treatment*:ab,ti OR therap*:ab,ti OR behavioural:ab,ti OR behavioral:ab,ti OR counsel*:ab,ti) | **48197** | **277,541** |
| **#1** | (ti:ab,ti AND (aged:ab,ti OR old*:ab,ti OR geriatric*:ab,ti OR aging:ab,ti OR ageing:ab,ti OR 'older adults':ab,ti OR older*:ab,ti OR elder*:ab,ti OR senior*:ab,ti OR geriatric:ab,ti OR 'late life':ab,ti OR 'old age':ab,ti OR elder$:ab,ti) OR ab:ab,ti) AND (aged:ab,ti OR old*:ab,ti OR geriatric*:ab,ti OR aging:ab,ti OR ageing:ab,ti OR 'older adults':ab,ti OR older*:ab,ti OR elder*:ab,ti OR senior*:ab,ti OR geriatric:ab,ti OR 'late life':ab,ti OR 'old age':ab,ti OR elder$:ab,ti) | **14056** | **145,545** |
