# Supplementary material for: The Impact of Psychosocial Interventions on Older Adults in the Community Experiencing Social Isolation: An Integrative Review
Source: Int J Ment Health Nurs. 2025 Dec 2;34(6):e70186. doi: 10.1111/inm.70186 (PMC12673195; doi:10.1111/inm.70186)
Supplement: Supplementary file 2 — File S2: Quality assessment. [file INM-34-0-s002.docx]

**Supplementary File 2: Quality Assessment**

| **Joanna Briggs Institute: Qualitative Research Checklist** | | Bantry-White et al. 2018 | Fakoya et al. 2023 | Lapena et al. 2020 | Hemingway et al. 2013 |
| --- | --- | --- | --- | --- | --- |
| 1 | Is there congruity between the stated philosophical perspective and the research methodology? | Yes | Yes | Yes | Yes |
| 2 | Is there congruity between the research methodology and the research question or objectives? | Yes | Yes | Yes | Yes |
| 3 | Is there congruity between the research methodology and the methods used to collect data? | Yes | Yes | Yes | Yes |
| 4 | Is there congruity between the research methodology and the representation and analysis of data? | Yes | Yes | Yes | Yes |
| 5 | Is there congruity between the research methodology and the interpretation of results? | Yes | Yes | Yes | Yes |
| 6 | Is there a statement locating the researcher culturally or theoretically? | No | No | No | No |
| 7 | Is the influence of the researcher on the research, and vice- versa, addressed? | No | No | No | No |
| 8 | Are participants, and their voices, adequately represented? | Yes | Yes | Yes | Yes |
| 9 | Is the research ethical according to current criteria or, for recent studies, and is there evidence of ethical approval by an appropriate body? | Yes | Yes | Yes | Yes |
| 10 | Do the conclusions drawn in the research report flow from the analysis, or interpretation, of the data? | Yes | Yes | Yes | Yes |

| **Joanna Briggs Institute: Quasi-Experimental Studies** | | Santos-Olmo et al. 2022 |
| --- | --- | --- |
| 1 | Is it clear in the study what is the ‘cause’ and what is the ‘effect’ (i.e. there is no confusion about which variable comes first)? | Yes |
| 2 | Were the participants included in any comparisons similar? | Yes |
| 3 | Were the participants included in any comparisons receiving similar treatment/care, other than the exposure or intervention of interest? | Yes |
| 4 | Was there a control group? | Yes |
| 5 | Were there multiple measurements of the outcome both pre and post the intervention/exposure? | Yes |
| 6 | Was follow up complete and if not, were differences between groups in terms of their follow up adequately described and analyzed? | Yes |
| 7 | Were the outcomes of participants included in any comparisons measured in the same way? | Yes |
| 8 | Were outcomes measured in a reliable way? | Yes |
| 9 | Was appropriate statistical analysis used? | Yes |
